# Intergenerational Mobility in Relative Educational Attainment and Health-Related Behaviours

**DOI:** 10.1007/s11205-017-1834-7

**Published:** 2018-01-05

**Authors:** Alexi Gugushvili, Martin McKee, Michael Murphy, Aytalina Azarova, Darja Irdam, Katarzyna Doniec, Lawrence King

**Affiliations:** 1 Department of Social Policy and Intervention and Nuffield College, University of Oxford, Barnett House, 32 Wellington Square, Oxford, OX1 2ER UK; 2 European Centre on Health of Societies in Transition, London School of Hygiene and Tropical Medicine, London, UK; 3 Department of Social Policy, London School of Economics and Political Science, London, UK; 4 Department of Sociology, University of Cambridge, Cambridge, UK

**Keywords:** Relative intergenerational mobility, Education, Binge drinking, Smoking, Demographic cohort study

## Abstract

Research on intergenerational social mobility and health-related behaviours yields mixed findings. Depending on the direction of mobility and the type of mechanisms involved, we can expect positive or negative association between intergenerational mobility and health-related behaviours. Using data from a retrospective cohort study, conducted in more than 100 towns across Belarus, Hungary and Russia, we fit multilevel mixed-effects Poisson regressions with two measures of health-related behaviours: binge drinking and smoking. The main explanatory variable, intergenerational educational mobility is operationalised in terms of relative intergenerational educational trajectories based on the prevalence of specified qualifications in parental and offspring generations. In each country the associations between intergenerational educational mobility, binge drinking and smoking was examined with incidence rate ratios and predicted probabilities, using multiply imputed dataset for missing data and controlling for important confounders of health-related behaviours. We find that intergenerational mobility in relative educational attainment has varying association with binge drinking and smoking and the strength and direction of these effects depend on the country of analysis, the mode of mobility, the gender of respondents and the type of health-related behaviour. Along with accumulation and Falling from Grace hypotheses of the consequences of intergenerational mobility, our findings suggest that upward educational mobility in certain instances might be linked to improved health-related behaviours.

## Introduction

For most of the post-war period, parents in western countries could expect their children to far better than they did. No longer. Data from the USA show that, while 90% of children born in the 1940s earned more than their parents, that figure is falling rapidly and is now only 50% for those born in the 1980s (Chetty et al. 2017). This has many implications for society, especially when combined with other factors, such as housing prices and pension changes, that mean that younger people now are accumulating wealth at a much lower level in the past (D’Arcy and Gardiner 2017). But what does it mean for health-related behaviours? There is a considerable body of evidence linking intergenerational social mobility with health outcomes, discussed further below (Boyle et al. 2009; Cardano et al. 2004; Dahl 1996; Faresjö et al. 1994; Hart et al. 1998, 2009; Jonsson et al. 2017; Padyab and Norberg 2014; Power et al. 1996).

One important pathway in these relationships, health-related behaviours, has not received much scholarly attention (Burrows and Nettleton 1995; Hemmingsson et al. 1999; Jefferis et al. 2004; Karvonen et al. 1999; Paavola et al. 2004). These are particularly salient in post-socialist societies where the transition has disrupted traditional intergenerational relationships (Gugushvili 2015, 2016a, b, 2017a; Lippényi and Gerber 2016; Titma and Roots 2006) and where certain health-related behaviours play a major role in population health (Bobak et al. 1999; Cockerham et al. 2006; Fiatal et al. 2016; Gilmore et al. 2001). While there is sociological research on the trends and correlates of intergenerational social mobility in post-socialist contexts and public health literature on the correlates of drinking and smoking, virtually no studies we are aware of enquire into the links between intergenerational social mobility and health-related behaviours.

The lack of empirical research itself justifies our study. However, another rationale stems from recent article by Campos-Matos and Kawachi (2015) in which they find that the association between intergenerational social mobility, assessed by educational attainment, and self-rated health varies among different welfare regimes in Europe. Their findings suggest that upward intergenerational mobility in the former Soviet Union and other post-socialist societies is associated with lower risk of reporting bad or very bad self-rated health when compared to those who remained intergenerationally non-mobile. Indeed, it was only in the former Soviet republics that the differences in self-related health between socially mobile and non-mobile groups were significant. Here we use an alternative data set, methods and operationalization of intergenerational educational mobility to explore possible pathways between intergenerational mobility and health, via their health-related behaviours such as binge drinking and smoking. We do so in three post-socialist societies, Belarus, Hungary and Russia.

## Theoretical Considerations

**Fig. 1:**
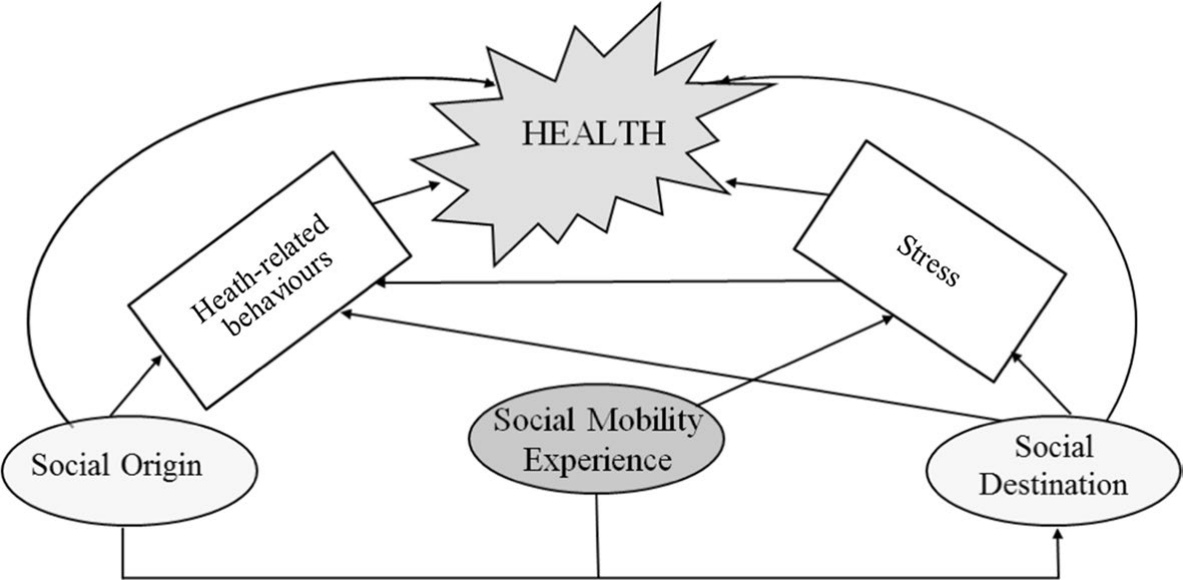
Theoretical model linking intergenerational mobility and individuals’ health. *Source*: Authors’ interpretation

There is no unified theory to suggest how intergenerational social mobility affects health-related behaviours as the studies testing the links between the two are virtually non-existent. Nonetheless, numerous scholars in sociology and public health have investigated health consequences of intergenerational social mobility experience (Heraclides and Brunner 2010; Houle and Martin 2011; Iveson and Deary 2017; Nicklett and Burgard 2009). Depending on the direction of mobility, upward versus downward, and the type of mechanisms involved, including accumulation of advantages or disadvantages, improvements in living standards, psychosocial effects and social learning, scholars expect to find positive or negative consequences of intergenerational mobility on health outcomes. Most existing hypotheses in this area of research emphasize the role of stress related to social mobility as the main causal channel affecting health of socially mobile individuals. On the other hand, it is well documented that stress among individuals is strongly related to their drinking and smoking patterns (Ayer et al. 2011; Childs and de Wit 2010; Kouvonen 2005; Steptoe et al. 1996; Zhang and Hwang 2007). This might suggest that intergenerational social mobility affects individuals’ stress levels which in turn could be related to health-related behaviours. Consequently, both stress and health-related behaviours might influence individuals’ health. These associations are illustrated in Fig. 1 which also shows that they are direct effects of individuals’ social origin and destination socioeconomic position on their health.

The links between intergenerational mobility and health-related behaviours can be also understood in terms of an accumulation model of health outcomes, which sees individuals’ health at any one time as the consequence of cumulative exposures to biological, psychosocial and material circumstances up to that time (Bartley and Plewis 2007). If we again refer to the links between intergenerational social mobility and health, numerous studies find that upwardly mobile individuals have better health than those in the social environment they leave behind but poorer health than those in the adult social environment they join (Blane et al. 1993; Boyle et al. 2009). The accumulation model implies that, after controlling for social origins, moving upward (downward) by virtue of social mobility reduces (increases) harmful exposures compared to non-mobile individuals, which in turn leads to better (worse) health outcomes. The same logic applies to individuals’ health-related behaviours. The large social gradient in hazardous drinking and smoking is well established (Marmot 2005) but these behaviours are, to varying degrees, conditioned by behaviours in early life (Jefferis et al. 2003). For instance, those who do not initiate smoking in adolescence are very unlikely to do so later (Giovino et al. 1995).

Although the accumulation perspective is an important mechanism, psychosocial effects are thought to be the main channel which links intergenerational social mobility and health-related behaviours. The dissociative thesis, probably the most widely tested hypothesis in the literature on consequences of social mobility, views moving away from one’s social origins as disruptive because mobile individuals have acquired their worldviews in one type of environment but must confront different sets of attitudes and values in another (Daenekindt 2016; Jonsson et al. 2017). Not being fully integrated in either social environment, these mobile individuals may feel less satisfied with life, experience increased distress, and consequently engage in negative health-related behaviours (Houle 2011). Linked to this is the Falling from Grace hypothesis (Newman 1999), whereby downward social mobility, in addition to any specific consequences of low socio-economic status, elevates levels of distress and feelings of insecurity as individuals find themselves less able to direct their own lives than they had expected when growing up which consequently also influences their health-related behaviours.

The main shortcoming of the dissociative and the Falling from Grace hypotheses is their prediction that both upward and downward intergenerational social mobility are associated with feelings of anxiety, strain, and distress without considering possible positive psychosocial outcomes of social mobility. Thus, several studies do not find significant associations between intergenerational mobility and health or even find that upward social mobility can lead to positive health-related behaviours and better health outcomes (Hemmingsson et al. 1999; Houle 2011). There are specific reasons why upwardly mobile individuals might have better health-related behaviours than comparable non-mobile groups. An improvement in socioeconomic status could have a protective effect on individuals’ psychological wellbeing by, for example, generating confidence and a sense of control of one’s own life (Poulton et al. 2002). It is also possible that upwardly mobile individuals might seek to distance themselves from their social origins by enthusiastically following the dominant lifestyle and health-related behaviours associated with their new social environment (Burrows and Nettleton 1995).

The aim of this article is to test which of the described alternative hypotheses most appropriately explains an association between intergenerational mobility and health-related behaviours, if there is any, in three post-socialist societies. We do this using a newly generated data set which includes information on respondents’ educational attainment and their drinking and smoking behaviours and that of their parents. In addition to investigating the association of intergenerational social mobility and health-related behaviours, this article also makes a methodological and conceptual contribution to the literature. First, recent social mobility scholarship increasingly recognises that, in order to understand the net effect of intergenerational educational mobility, individuals’ and their parents education has to be viewed and operationalised in relative rather than absolute terms, that is, as a positional good, taking into account the relative prevalence of qualifications in parental and offspring generations (Goldthorpe 2014; Gugushvili et al. 2017). Second, most existing studies in public health and social epidemiology look at the implications of intergenerational social mobility on health-related behaviours of young people, while social mobility literature suggests that individuals only achieve a mature socio-economic status later in life (Bukodi and Goldthorpe 2016). Our dataset, described in detail in the next section, consists of individuals aged 40 and above, offering an advantage over existing studies which examine social mobility among those who have yet to reach maturity in their socio-economic status.

## Methods

### Dataset

Our analyses are based on the PrivMort data set, collected within a multi-disciplinary project funded by the European Research Council whose main objective is to investigate the post-Communist morbidity and mortality crisis using a multi-level retrospective cohort study (Irdam et al. 2016). PrivMort data are from original surveys in 20, 52 and 30 towns respectively in Belarus, Hungary and the European part of Russia. We conducted surveys in Belarus and the European part of Russia mainly because the post-Communist health and mortality crisis was especially severe in these regions of the former Soviet Union. Belarus and most of European Russia (see below) have relatively homogeneous populations, sharing similar socio-economic, cultural and religious characteristics, including infamously high alcohol and tobacco consumption, especially among men (Cockerham 2000; Gilmore et al. 2001; Pomerleau et al. 2008). However, the two countries have experienced a different pace and type of transition. On the other hand, Hungary allows investigation of the transitional health crisis in a non-Soviet post-communist country. Like in Belarus and Russia, life expectancy in Hungary declined significantly at the beginning of the transition and the country also has a high level of alcohol consumption, particularly home-distilled liquors (pálinka) (Szucs et al. 2005), as well as high levels of smoking (Forey et al. 2013).

During the first stage of the project, PrivMort collected basic economic, demographic and enterprise-level data on all towns of Russia with 5000–100,000 inhabitants in the European part of the country, excluding the regions of the North Caucasus. These regions were excluded as some have substantial Muslim populations, with dietary and alcohol consumption very different from the rest of Russia, which might potentially confound health outcomes. A set of 30 towns was selected from the pool of 539 using propensity score matching for the likelihood of experiencing rapid privatization. Propensity scores were calculated on the basis of the pre-transition demographic and socio-economic conditions in the towns. Since one of the main goals of PrivMort project was to identify the health consequences of rapid privatisation, ten mono-industrial towns (defined as having a single industrial enterprise providing employment for at least 7.5% of the total population) with rapid privatization (where 90 or more per cent of state shares were privatized within two consecutive years) were matched to ten mono-industrial towns with slow privatization (where less than 50% of state shares were privatized within two consecutive years). Additionally, a group of five multi-industrial towns (where employment is distributed proportionally among several industrial enterprises) was selected closely matching an additional five mono-industrial towns.

In Belarus, as in Russia, only towns with 5000–100,000 inhabitants were included. Out of the pool of 96 towns which fall into this category, all 11 mono-towns were selected for survey data collection. Then, 9 multi-slow towns in Belarus were matched to existing fifteen mono-fast towns in Russia. In Hungary, we selected all towns with 5000–100,000 inhabitants where industrial employment exceeded 30% at the beginning of the 1990s. As in the Russian and Belarusian samples, towns within commuting distance to the capital were excluded. 52 towns were randomly selected from a total sample of 83 towns in Hungary matching the selection criteria.

In the selected towns, houses/apartments were randomly chosen using the random walk method between June 2014 and April 2015, and interviewers of survey organisations VCIOM, SIMST and TARKI in Russia, Belarus and Hungary respectively, conducted face-to-face interviews. Only one respondent was selected from each household. To be included, a potential respondent had to be born before 1972, and have at least one family member (parents, siblings, or husbands) who lived in the same town for a prolonged period of time during and after the transition. This criterion ensured that a respondent had reached working age by 1991. Overall 16,000, 24,072 and 24,069 individuals were interviewed in Belarus, Hungary and Russia. In addition to information collected on respondents’ socio-demographic, socio-economic and health-related behaviours, PrivMort acquired data on their fathers’ and mothers’ educational attainment, used to derive the intergenerational educational mobility variable employed in our analysis of respondents’ likelihood to binge drink and smoke.

To compensate for missing data on paternal education, which was not reported by some respondents (13.7, 16.6 and in 19.5%, respectively, in Belarus, Hungary and Russia) we used multiple imputation via the MICE (Multiple Imputation using Chained Equations) package in Stata, version 14, allowing for twenty sets of multiple imputations and combining them using Rubin’s (1987) rules. Although we stratify our analyses by gender, in all countries women are overrepresented in the data set—76.3% in Belarus, 65.8% in Hungary and 77.8% in Russia. The latter is a result of significantly more men being away from their households as well as men having higher mortality rates than women in these countries (Gugushvili 2016a). More details concerning the selection of towns, respondents and other aspects of the PrivMort methodology are given elsewhere (Azarova et al. 2017; Irdam et al. 2016).

### Outcome Variables

We use two measures of health-related behaviour as our outcome variables—binge drinking and smoking. Binge drinking is operationalised as a dummy variable which takes value of 1 if respondents’ drink up to half a litre of vodka (in Russia and Belarus) or brandy ((pálinka) in Hungary) or two bottles of wine or 5 bottles of beer in one evening at least once a month or more often. Our dummy variable for smoking captures those individuals who report being regular smokers as opposed to individuals who have never smoked or used to smoke but have quit. Table 1 shows the frequencies of binge drinking and smoking across Belarus, Hungary and Russia. In the post-Soviet countries about one-fourth of men report binge drinking in the past month, while in Hungary this is about 13%. Binge drinking among women is also more prevalent in Russia (5.2%) and Belarus (6.6%) than in Hungary (2.2%). Russia and Belarus are very similar in terms of the prevalence of current smokers among men (about 40%), while Hungary has much higher rate of smoking among women (15.2%) than Belarus (3.6%) and Russia (4.5%).

**Table 1:** Descriptive statistics of dependent variables, %. *Source*: Authors’ calculations based on the PrivMort data set

	Men			Women		
	Belarus	Hungary	Russia	Belarus	Hungary	Russia
Binge drinking						
Yes	24.5	12.5	26.4	6.6	2.2	5.2
No	75.5	87.5	73.6	93.4	97.8	94.8
In totals	100.0	100.0	100.0	100.0	100.0	100.0
Smoking						
Yes	40.3	29.4	42.9	3.6	15.2	4.5
No	59.7	70.6	57.1	96.4	84.8	95.5
In totals	100.0	100.0	100.0	100.0	100.0	100.0

### Intergenerational Educational Mobility

In order to generate the main independent variable used in this study, intergenerational mobility in educational status, we use the PrivMort variables on the highest level of education respondents and their parents attained. In both instances the level of education is classified into eight categories: (a) incomplete elementary; (b) complete elementary or incomplete secondary; (c) complete academic secondary; (d) complete vocational secondary without general high school leaving exam; (e) complete vocational secondary with general high school leaving exam; (f) incomplete higher; (g) complete vocational higher; and (h) complete academic higher.

The most straightforward way of generating a variable of intergenerational educational mobility would be to compare the formal level of educational attainment between parents and their children, but this approach would ignore the continuous improvement over time of levels of education in socialist and post-socialist societies (Gerber and Hout 1995; Higher Education in Europe 1977) and its implications for inequality (Raftery and Hout 1993; Shkolnikov 2006).

Education, like other markers of status, is believed to be a positional good, which means that the value of educational qualifications diminishes in proportion to the number of individuals who acquire them (Breen et al. 2009). In addition, if education serves as one of the main characteristics for employers in selecting employees, the quality of jobs that are available to individuals will depend not only on how much education they have acquired, but also on how well educated they are relative to others in the labour pool (Goldthorpe 2014). In terms of health-related behaviours, subjective socio-economic status has been shown to be an important covariate of drinking and smoking (Reitzel et al. 2010; Ritterman et al. 2009). Therefore, intergenerational educational mobility in relative terms could be more appropriate than absolute educational mobility in capturing intergenerational changes in position in social hierarchy and the resultant consequences for health-related behaviours.

To derive the measure of intergenerational educational mobility, we generate relative educational attainment separately for fathers and mothers by creating the approximate tertiles of relative educational level with the following two-step procedure (see Gugushvili et al. 2017). First, based on the distribution of attained education in each of the following birth-cohorts—born before 1905, in 1906–1915, 1916–1925, 1926–1935, 1936–1945 and 1946–1954—we divide education into approximate tertiles in each group. Second, we use these variables to generate a single measure of educational attainment shown in Table 2, employing the “dominance” method, which defines the indicator of parental education as the highest level of father or mother (Erikson 2006).

**Table 2:** Relative education tertiles of parents and respondents, %. *Source*: Authors’ calculations based on the PrivMort data set

Tertile	Belarus		Hungary		Russia	
	Parents	Respondents	Parents	Respondents	Parents	Respondents
Men						
Bottom	52.3	46.3	45.5	47.0	47.7	43.8
Middle	17.0	33.6	29.6	31.7	21.1	39.0
Top	30.7	20.1	24.9	21.2	31.3	17.2
In total	100.0	100.0	100.0	100.0	100.0	100.0
Women						
Bottom	66.0	46.6	51.3	63.3	57.1	43.5
Middle	13.0	35.2	28.9	21.0	19.0	41.6
Top	21.0	18.2	19.8	15.7	23.9	15.0
In total	100.0	100.0	100.0	100.0	100.0	100.0

For respondents’ educational attainment we use the same procedure as described for their parents, but in this case relative tertiles are calculated separately for five age groups—40–50, 51–60, 61–70, 71–80 and more than 80. The distribution of tertile frequencies for respondents are shown in Table 2. In all countries, the largest tertiles are the bottom ones, while about 20% of respondents are in the top educational tertiles. The latter can be explained by the higher prevalence of primary and vocational education in comparison to tertiary education in the Belarus, Hungary and Russia.

**Fig. 2:**
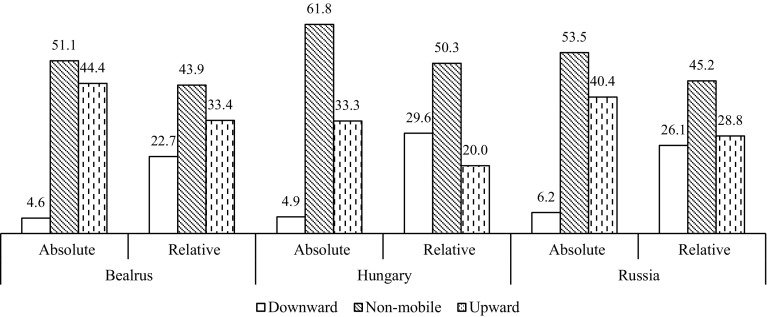
Comparing absolute and relative intergenerational educational mobility in Belarus, Hungary and Russia, %. *Source*: Authors’ calculations based on the PrivMort data set

In Fig. 2 we classify respondents as upwardly (downwardly) mobile if they are in a higher (lower) tertile of education attainment than their parents; the remaining respondents are treated as non-mobile. For brevity of presentation, we pool data for both genders and compare our relative education mobility measure with the absolute education mobility measure. The latter is based on the simple comparison of respondents’ and their parents’ formal educational attainment without considering the relative prevalence of these qualifications. It is clear that in all three countries substantially more individuals attained higher levels of formal education than their parents—more than two-fifths in Belarus and Russia and one-third in Hungary. However, when it comes to relative mobility, upwardly mobile, downwardly mobile and non-mobile individuals are much more equally distributed. The lowest mobility is observed in Hungary which is in line with the recent findings on the rigidity of intergenerational status reproduction in this country (Bukodi et al. 2017; Gugushvili 2017b).

### Analytic Strategy

In order to detect the effect of intergenerational educational mobility, we identify those respondents in the bottom, the middle and the top educational tertiles who are upwardly mobile, downwardly mobile and non-mobile. We investigate how far specific mobility trajectories are associated with binge drinking and smoking. For this purpose, we construct an educational mobility variable by cross-tabulating the respondents’ and their parents’ educational attainment in nine trajectories (see Li et al. 2008): (1) the stable bottom educational tertile, (2) downwardly mobile from the middle to the bottom educational tertile; (3) downwardly mobile from the top to the bottom educational tertile; (4) upwardly mobile from the bottom to the middle educational tertile; (5) the stable middle educational tertile; (6) downwardly mobile from the top to the middle educational tertile; (7) upwardly mobile from the bottom to the top educational tertile; (8) upwardly mobile from the middle to the top educational tertile; (9) the stable top educational tertile. As Table 3 indicates the largest group of respondents are non-mobile individuals in the bottom educational tertile, but upwardly mobile individuals in the middle educational tertile also constitute a sizable mobility trajectory in all considered societies.

**Table 3:** Intergenerational educational trajectories in Belarus, Hungary and Russia, %. *Source*: Authors’ calculations based on the PrivMort data set

Mobility trajectories	Men			Women		
	Belarus	Hungary	Russia	Belarus	Hungary	Russia
Bottom tertile						
Parent 1st → respondent 1st	26.5	27.4	26.1	34.4	38.8	30.8
Parent 2nd → respondent 1st	8.9	12.9	8.8	5.6	17.2	6.8
Parent 3rd → respondent 1st	11.0	6.7	8.9	6.6	7.2	5.8
Middle tertile						
Parent 1st → respondent 2nd	18.4	13.1	16.5	22.6	8.5	21.1
Parent 2nd → respondent 2nd	4.4	10.1	8.6	4.4	6.6	8.7
Parent 3rd → respondent 2nd	10.6	8.6	13.9	8.1	5.9	11.8
Top tertile						
Parent 1st → respondent 3rd	7.5	5.0	5.1	9.0	4.1	5.3
Parent 2nd → respondent 3rd	3.6	6.6	3.7	3.0	5.0	3.5
Parent 3rd → respondent 3rd	9.1	9.6	8.5	6.3	6.6	6.3
In total	100.0	100.0	100.0	100.0	100.0	100.0

Our analytical strategy implies that when the results are reported as incidence rate ratios the reference categories include non-mobile individuals in the bottom, the middle and the top educational tertiles. In practice, this means that we run three separate regressions each time, changing only the reference category which does not affect the overall model fit or the relative values of the regression coefficients for other variables included in the models. In the remainder of this article, we use terms “intergenerational educational mobility” and “educational trajectories” interchangeably. To disentangle the effect of intergenerational educational mobility from parental and respondents’ own attainment, we could alternatively use diagonal reference models (Daenekindt 2016; Houle and Martin 2011; Liang and Lu 2014), but it has been demonstrated that the latter approach produces very similar results to those derived from intergenerational mobility trajectories (Chan 2017). In addition, the substantive interpretation of diagonal reference models is less straightforward than identifying the consequences of mobility for individuals with different intergenerational trajectories.

Since both of our dependent variables have a binary form, for each country we fit two-level random-intercept mixed-effects Poisson regressions using the “mepoisson” command in the statistical software Stata, version 14. In this hierarchical model specification, which is widely used in social science research, we identify the group structure for the random effects at the town level separately in Belarus, Hungary and Russia. We do not include specific contextual variables on the town level primarily because of comparability of measures derived from different statistical sources. We run models separately for men and women as there are substantial differences in drinking and smoking in the considered societies. We also calculate post-estimation predicted probabilities of binge drinking and smoking with corresponding 95% CIs for individuals in all nine mobility trajectories (King et al. 2012).

### Covariates

Our regression models, in addition to accounting for intergenerational mobility in educational attainment and corresponding levels of parental and individuals’ education, control for important covariates which have been identified in the previous research to be associated with binge drinking and smoking (Burrows and Nettleton 1995; Cockerham et al. 2006; Hart et al. 1998; Hemmingsson et al. 1999; Jefferis et al. 2004; Johnson et al. 2010; Levin 1994; Paavola et al. 2004; Pomerleau et al. 2004; Tumen and Zeydanli 2014), as shown in Table 4. Respondents’ age is categorized into five groups: 40–50, 51–60, 61–70, 71–80, and those older than 80. Marital status is operationalised with single, married, separated/divorced and widowed individuals. To account for socio-economic status we consider private ownership of a house/flat and a car by the household where respondents live. The latter two variables take a value of 3 if their households owned a house/flat or a car throughout three decades in the 1980s, 1990s, and 2000s and a value 0 if not owned in the corresponding periods. Respondents’ labour market status is operationalised by being in or out of work at interview. We account for individuals’ supervisory responsibilities and self-employment/entrepreneurship status in the 1990s–2000s. Lastly, we also control for respondents’ religious denomination as some religions sanction particular behaviours related to alcohol and tobacco consumption. Descriptive statistics indicates that for most of the control variables Belarus and Russia are similar to each other, while Hungary stands out with its higher ownership of cars and private apartments and its lower prevalence of working individuals and those with supervisory status.

**Table 4:** Descriptive statistics of independent variables, %. *Source*: Authors’ calculations based on the PrivMort data set

	Men			Women		
	Belarus	Hungary	Russia	Belarus	Hungary	Russia
Respondents’ age						
40–50	30.5	20.9	26.7	19.8	16.3	18.0
51–60	30.1	24.0	31.6	27.8	22.8	27.3
61–70	22.2	26.5	25.1	25.3	28.4	28.6
71–80	12.1	20.1	12.6	19.7	23.2	19.6
80 and over	5.1	8.5	4.0	7.5	9.3	6.6
Total	100.0	100.0	100.0	100.0	100.0	100.0
Marital status						
Single	5.0	8.7	6.4	2.5	3.9	3.8
Married	69.6	60.4	70.6	50.6	46.8	50.3
Separated/divorced	14.7	15.8	12.5	11.9	14.1	11.8
Widow/widower	10.8	15.2	10.5	35.0	35.2	34.2
In total	100.0	100.0	100.0	100.0	100.0	100.0
Owning house/flat in 1980s–2000s						
Not at all	5.4	1.8	6.1	4.5	2.2	6.1
Only in one decade	4.7	1.6	2.4	3.3	1.5	2.8
In two decades	11.7	4.6	8.8	11.6	4.8	9.4
Throughout	78.2	92.1	82.7	80.5	91.5	81.8
In total	100.0	100.0	100.0	100.0	100.0	100.0
Owning car in 1980s–2000s						
Not at all	39.9	34.3	44.6	58.2	45.9	62.2
Only in one decade	17.6	9.0	15.9	13.7	9.7	12.6
In two decades	21.3	16.2	18.0	14.6	14.5	12.3
Throughout	21.2	40.5	21.5	13.5	29.9	12.9
In total	100.0	100.0	100.0	100.0	100.0	100.0
Work status						
Working	55.6	37.6	49.6	37.4	27.0	35.3
Not working	44.4	62.4	50.4	62.6	73.0	64.7
In total	100.0	100.0	100.0	100.0	100.0	100.0
Supervisory responsibilities						
Yes	24.3	9.6	23.5	19.3	6.6	19.7
No	75.7	90.4	76.5	80.7	93.4	80.3
In total	100.0	100.0	100.0	100.0	100.0	100.0
Self-employment						
Yes	6.6	3.7	5.8	3.0	1.9	2.8
No	93.4	96.3	94.3	97.1	98.1	97.2
In total	100.0	100.0	100.0	100.0	100.0	100.0
Religious denomination						
Orthodox	83.3	–	89.3	84.9	–	95.0
Non-Orthodox Christian	11.7	84.9	1.1	13.7	90.7	1.0
Muslim	0.3	–	1.1	0.1	–	1.2
Other	4.7	15.1	8.5	1.3	9.3	2.8
In total	100.0	100.0	100.0	100.0	100.0	100.0

## Results

### Baseline Analysis

The results presented in Tables 5 and 6 are adjusted for respondents’ age and indicate that for both genders the standard deviation of the intercepts across towns in Belarus, Hungary and Russia is higher for binge drinking than is the case for smoking. This suggests that towns where respondents were interviewed are less similar to each other in terms of binge drinking than they are in terms of smoking. The interclass correlation coefficients (ICC) also show that nesting individuals in towns account for up to 12 and 20% variance in binge drinking, respectively for women and men even after controlling for individual-level predictors, while for smoking the ICC is less than 5%, except for women in Russia where the value is 9%.

**Table 5:** Intergenerational educational mobility, binge drinking and smoking among men. *Source*: Authors’ calculations based on the PrivMort data set

	Binge drinking			Smoking		
	Belarus	Hungary	Russia	Belarus	Hungary	Russia
Mobility trajectories						
Bottom (ref: non-mobile in 1st tertile)						
Parent 2nd → respondent 1st	0.97	**0.83**	1.05	0.98	0.91	1.05
	(0.85, 1.10)	**(0.71, 0.98)**	(0.91, 1.22)	(0.89, 1.08)	(0.82, 1.01)	(0.96, 1.15)
Parent 3rd → respondent 1st	**0.80**	**0.74**	1.15	0.97	0.92	1.03
	**(0.66, 0.96)**	**(0.60, 0.92)**	(0.93, 1.43)	(0.88, 1.07)	(0.82, 1.04)	(0.94, 1.14)
Middle (ref: non-mobile in 2nd tertile)						
Parent 1st → respondent 2nd	1.13	**1.36**	0.92	0.94	0.97	0.96
	(0.87, 1.48)	**(1.06, 1.73)**	(0.74, 1.14)	(0.78, 1.14)	(0.84, 1.11)	(0.87, 1.06)
Parent 3rd → respondent 2nd	1.03	1.09	1.16	0.99	0.90	1.08
	(0.78, 1.37)	(0.81, 1.48)	(0.90, 1.49)	(0.79, 1.23)	(0.75, 1.09)	(0.96, 1.22)
Top (ref: non-mobile in 3rd tertile)						
Parent 1st → respondent 3rd	0.88	1.04	0.75	**0.70**	0.96	0.75
	(0.63, 1.22)	(0.68, 1.59)	(0.49, 1.15)	**(0.53, 0.93)**	(0.69, 1.32)	(0.55, 1.02)
Parent 2nd → respondent 3rd	0.92	0.99	0.98	0.87	1.08	0.92
	(0.68, 1.23)	(0.65, 1.50)	(0.78, 1.23)	(0.66, 1.16)	(0.84, 1.41)	(0.74, 1.14)
Marital status (ref: married)						
Single	1.10	1.18	0.94	1.04	1.09	0.91
	(0.89, 1.37)	(0.96, 1.46)	(0.78, 1.13)	(0.95, 1.14)	(0.96, 1.23)	(0.81, 1.03)
Separated	**1.29**	**1.51**	**1.16**	**1.24**	**1.31**	**1.10**
	**(1.15, 1.45)**	**(1.32, 1.74)**	**(1.00, 1.34)**	**(1.14, 1.36)**	**(1.22, 1.41)**	**(1.02, 1.18)**
Widow/widower	0.92	1.08	0.94	1.02	1.11	0.95
	(0.73, 1.16)	(0.87, 1.33)	(0.82, 1.08)	(0.88, 1.18)	(0.98, 1.25)	(0.84, 1.08)
Wealth						
Owing a house	0.96	**0.87**	0.97	1.03	**0.92**	1.02
	(0.87, 1.06)	**(0.80, 0.94)**	(0.86, 1.09)	(0.98, 1.09)	**(0.88, 0.96)**	(0.99, 1.06)
Owing a car	0.99	0.98	0.95	0.99	**0.89**	**0.96**
	(0.94, 1.04)	(0.92, 1.03)	(0.89, 1.02)	(0.97, 1.01)	**(0.86, 0.92)**	**(0.94, 0.99)**
Labour market						
Working (ref: not working)	0.95	0.91	0.96	**0.87**	**0.88**	0.95
	(0.79, 1.14)	(0.80, 1.04)	(0.83, 1.12)	**(0.79, 0.96)**	**(0.81, 0.95)**	(0.87, 1.04)
Supervisor status (ref: no)	0.99	1.13	0.88	0.94	0.86	**0.86**
	(0.83, 1.18)	(0.89, 1.43)	(0.77, 1.01)	(0.89, 1.00)	(0.74, 1.00)	**(0.80, 0.93)**
Self-employed (ref: no)	1.03	1.00	1.08	**0.85**	0.95	1.04
	(0.84, 1.25)	(0.75, 1.31)	(0.90, 1.28)	**(0.76, 0.95)**	(0.78, 1.15)	(0.96, 1.13)
Religion						
Other Christian (ref: Orthodox)	0.97	–	0.81	0.89	–	0.74
	(0.84, 1.13)	–	(0.55, 1.18)	(0.80, 1.00)	–	(0.51, 1.07)
Muslim	0.64	–	0.79	0.70	–	0.97
	(0.22, 1.87)	–	(0.58, 1.09)	(0.37, 1.33)	–	(0.78, 1.22)
Other	0.88	**1.16**	1.05	0.90	**1.11**	1.08
	(0.70, 1.10)	**(1.00, 1.35)**	(0.89, 1.24)	(0.75, 1.07)	**(1.03, 1.21)**	(0.98, 1.20)
Intercept	**0.13**	**0.15**	**0.17**	**0.15**	**0.20**	**0.24**
	**(0.07, 0.24)**	**(0.11, 0.22)**	**(0.10, 0.27)**	**(0.10, 0.22)**	**(0.16, 0.25)**	**(0.18, 0.32)**
Random intercept	**0.18**	**0.12**	**0.11**	0.01	**0.03**	0.01
	**(0.05, 0.32)**	**(0.07, 0.17)**	**(0.04, 0.18)**	(− 0.00, 0.01)	**(0.01, 0.05)**	(− 0.00, 0.02)
ICC	0.12	0.06	0.07	0.01	0.03	0.02
Total observations/towns	4082/20	8457/52	6356/30	4082/20	8457/52	6356/30

Incidence rate ratios from multilevel mixed-effects Poisson regressions. For each country and health-related behaviour three separate models are fitted by changing reference category to non-mobile individuals in the bottom, middle and the top educational tertile. 95% CIs are in parentheses, significant associations are shown in bold

**Table 6:** Intergenerational educational mobility, binge drinking and smoking among women. *Source*: Authors’ calculations based on the PrivMort data set

	Binge drinking			Smoking		
	Belarus	Hungary	Russia	Belarus	Hungary	Russia
Mobility trajectories						
Bottom (ref: non-mobile in 1st tertile)						
Parent 2nd → respondent 1st	1.05	1.15	**1.44**	**1.41**	**0.89**	1.20
	(0.79, 1.41)	(0.84, 1.57)	**(1.18, 1.76)**	**(1.09, 1.82)**	**(0.81, 0.99)**	(0.98, 1.47)
Parent 3rd → respondent 1st	0.91	0.87	0.91	**1.22**	0.92	1.12
	(0.69, 1.20)	(0.52, 1.45)	(0.69, 1.20)	**(1.00, 1.48)**	(0.81, 1.03)	(0.90, 1.41)
Middle (ref: non-mobile in 2nd tertile)						
Parent 1st → respondent 2nd	1.11	1.15	1.01	0.75	1.14	0.85
	(0.75, 1.65)	(0.69, 1.92)	(0.78, 1.30)	(0.41, 1.38)	(0.97, 1.32)	(0.68, 1.06)
Parent 3rd → respondent 2nd	1.02	1.33	1.23	1.22	**1.17**	1.19
	(0.70, 1.49)	(0.80, 2.21)	(0.93, 1.63)	(0.70, 2.15)	**(1.00, 1.38)**	(0.91, 1.54)
Top (ref: non-mobile in 3rd tertile)						
Parent 1st → respondent 3rd	1.27	0.81	1.05	0.83	1.14	1.03
	(0.94, 1.72)	(0.40, 1.62)	(0.73, 1.51)	(0.46, 1.51)	(0.90, 1.45)	(0.66, 1.63)
Parent 2nd → respondent 3rd	1.17	1.28	1.05	0.67	**1.28**	0.96
	(0.65, 2.11)	(0.72, 2.30)	(0.73, 1.52)	(0.43, 1.05)	**(1.03, 1.58)**	(0.65, 1.40)
Marital status (ref: married)						
Single	1.07	1.22	**1.25**	**1.93**	**1.20**	**1.73**
	(0.86, 1.32)	(0.90, 1.65)	**(1.00, 1.55)**	**(1.30, 2.86)**	**(1.03, 1.40)**	**(1.36, 2.19)**
Separated	1.12	1.17	1.04	**1.87**	**1.39**	**1.64**
	(0.93, 1.35)	(0.88, 1.56)	(0.89, 1.21)	**(1.59, 2.21)**	**(1.28, 1.50)**	**(1.39, 1.95)**
Widow/widower	0.94	0.72	0.98	**1.56**	1.07	**1.45**
	(0.76, 1.16)	(0.47, 1.09)	(0.84, 1.16)	**(1.28, 1.88)**	(0.97, 1.19)	**(1.23, 1.71)**
Wealth						
Owing a house	**0.81**	**0.83**	0.91	0.95	**0.85**	0.93
	**(0.67, 0.99)**	**(0.73, 0.93)**	(0.83, 1.00)	(0.87, 1.02)	**(0.82, 0.88)**	(0.87, 1.00)
Owing a car	1.04	0.96	1.03	0.95	**0.93**	**0.92**
	(0.97, 1.12)	(0.88, 1.04)	(0.96, 1.09)	(0.90, 1.01)	**(0.91, 0.96)**	**(0.87, 0.98)**
Labour market						
Working (ref: not working)	1.07	1.27	1.00	0.81	0.97	1.01
	(0.88, 1.30)	(0.89, 1.81)	(0.83, 1.20)	(0.66, 1.00)	(0.90, 1.04)	(0.80, 1.27)
Supervisor status (ref: no)	0.96	**1.69**	0.87	1.09	0.92	1.04
	(0.84, 1.09)	**(1.21, 2.38)**	(0.73, 1.03)	(0.91, 1.31)	(0.78, 1.08)	(0.90, 1.21)
Self-employed (ref: no)	1.14	1.16	1.16	1.70	1.17	**1.32**
	(0.79, 1.65)	(0.45, 3.01)	(0.82, 1.64)	(1.27, 2.28)	(0.92, 1.49)	**(1.00, 1.74)**
Religion						
Other Christian (ref: Orthodox)	0.93	–	0.73	1.04	–	1.10
	(0.70, 1.24)	–	(0.25, 2.16)	(0.86, 1.27)	–	(0.67, 1.81)
Muslim	0.00	–	0.64	0.88	–	1.36
	(0.00, 0.00)	–	(0.37, 1.08)	(0.44, 1.76)	–	(0.75, 2.47)
Other	0.74	**1.36**	1.08	0.94	**1.17**	**1.49**
	(0.42, 1.28)	**(1.04, 1.80)**	(0.70, 1.66)	(0.63, 1.39)	**(1.05, 1.31)**	**(1.14, 1.93)**
Intercept	**0.06**	**0.02**	**0.04**	**0.00**	**0.05**	**0.00**
	**(0.03, 0.13)**	**(0.01, 0.05)**	**(0.02, 0.06)**	**(0.00, 0.01)**	**(0.03, 0.08)**	**(0.00, 0.01)**
Random intercept	0.67	**0.58**	**0.52**	**0.12**	**0.03**	**0.26**
	(− 0.05, 1.39)	**(0.27, 0.90)**	**(0.24, 0.80)**	**(0.01, 0.22)**	**(0.01, 0.05)**	**(0.03, 0.48)**
ICC	0.20	0.16	0.16	0.04	0.02	0.09
Total observations/towns	11,918/20	15,615/52	17,713/30	11,918/20	15,615/52	17,713/30

Incidence rate ratios from multilevel mixed-effects Poisson regressions. For each country and health-related behaviour three separate models are fitted by changing reference category to non-mobile individuals in the bottom, middle and the top educational tertile. 95% CIs are in parentheses, significant associations are shown in bold

Before discussing the implications of social mobility for binge drinking and smoking, we briefly review the results for our confounding variables. As regards marital status’ association with the dependent variables, separated and divorced men have consistently higher incidence rate ratios of engaging in both negative health-related behaviours, while single and widowed women are also more likely to be current smokers. For men, living in their privately owned apartment/house in the 1980s–2000s reduces the risk of binge drinking and smoking only in Hungary, while among women it is associated with a reduced likelihood of binge drinking in all settings. For both men and women, having a car is associated with reduced smoking in Hungary and Russia. Working women in Belarus and working men in Belarus and Hungary are significantly less likely to smoke. Supervisory status is associated with higher risk of binge drinking among Hungarian women and lower risk of smoking among men in all included countries. Self-employment is related to lower levels of smoking among Belarusian men, but higher levels among Belarusian and Russian women. Among non-Christian Hungarians, men appear to have both higher incidence rate ratios of binge drinking and smoking, while non-Orthodox Christian men in Belarus have lower risk of smoking than Orthodox Christians. Lastly, non-Christian Hungarian women have higher likelihood of negative health-related behaviours and non-Christian women in Russia are also more likely to smoke.

Moving to the findings related to intergenerational educational mobility, results from multilevel mixed-effects Poisson regressions in Tables 5 and 6 demonstrate that, controlling for other confounders, intergenerational educational mobility does not have consistent associations with the health-related behaviours in all countries, although intergenerationally mobile individuals do, in some instances, differ from non-mobile individuals. Men in the bottom tertile of educational attainment whose parents were in the top educational tertile are less likely to binge drink in Belarus (IRR 0.80; 95% CI 0.66, 0.96) and Hungary (IRR 0.74; 95% CI 0.60, 0.92) when compared with intergenerationally non-mobile individuals with a low level of education. In addition, in Hungary, the incidence rate ratio of binge drinking is 0.83 (95% CI 0.71, 0.98) for downwardly mobile men from the middle to the bottom tertile of educational attainment. The latter findings tend to corroborate the accumulation hypothesis on social mobility and health-related behaviours than the Falling from Grace hypothesis. We observe the opposite relationship when looking at women in Russia. Those who are downwardly mobile, from the middle to the bottom educational tertile, have an incidence rate ratio of 1.44 (95% CI 1.18, 1.76) for binge drinking. This effect is the only significant association that we observe among women for binge drinking and it is in line with the Falling from Grace hypothesis on the negative consequences associated with downward mobility.

Significant associations are also found when considering the links between intergenerational educational mobility and smoking. Upwardly mobile Belarussian men from the lowest to the highest educational tertile are significantly less likely to smoke (OR 0.70 95% CI 0.53, 0.93) than non-mobile individuals with the same level of education. The latter is at odds with the dissociative thesis and instead supports a positive effect of upward social mobility on individuals’ health-related behaviours. Among women in Belarus downward mobility from the top and the middle educational tertiles in the bottom educational tertile is associated, respectively, with incidence rate ratios of 1.22 (95% CI 1.00, 1.48) and 1.41 (95% CI 1.09, 1.82) for being current smokers. This finding supports the prediction of negative consequences from downward intergenerational mobility. On the other hand, for Hungarian women the findings are more consistent with the accumulation hypothesis. The results suggest that both those who experience downward mobility to the bottom educational tertile from the middle one are less likely to be current smokers, with incidence rate ratios of 0.89 (95% CI 0.81, 0.99), while those who experience upward mobility to the top educational tertile from the middle one are more likely to be current smokers with incidence rate ratios of 1.28 (95% CI 1.03, 1.58).

### Predicted Probabilities

**Fig. 3:**
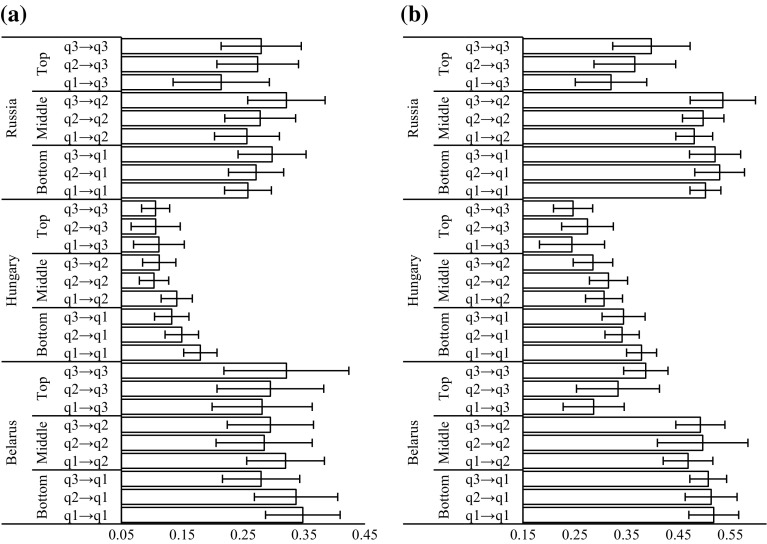
Predicted probabilities of **a** binge drinking and **b** smoking among men. *Notes* Error bars represent 95% CIs. qX → qX represent intergenerational trajectories from parental educational tertile to respondents educational tertile. *Source*: Authors’ calculations based on the PrivMort data set

**Fig. 4:**
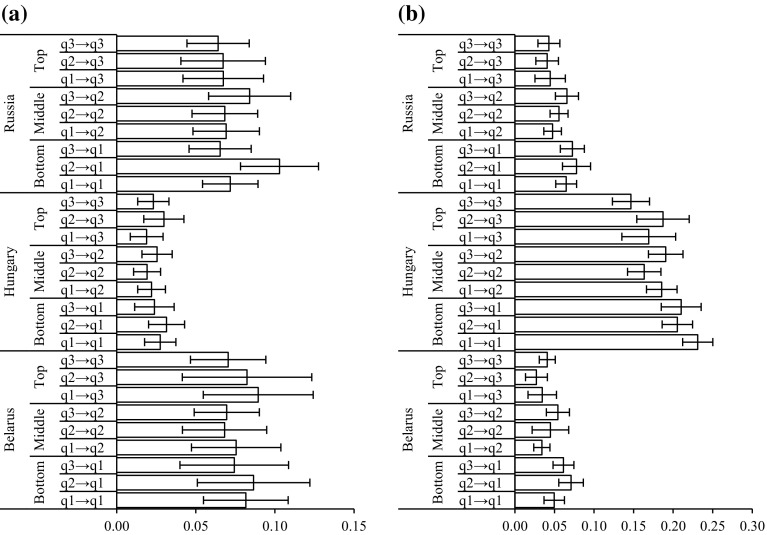
Predicted probabilities of **a** binge drinking and **b** smoking among women. *Notes* Error bars represent 95% CIs. qX → qX represent intergenerational trajectories from parental educational tertile to respondents educational tertile. *Source*: Authors’ calculations based on the PrivMort data set

As suggested in earlier research, findings on inequalities in health should be reported using both relative and absolute measures as presenting only one could mislead as to the magnitude, direction and significance of results (Campos-Matos and Kawachi 2015; King et al. 2012). Figures 3 and 4 show predicted probabilities and corresponding 95% CIs for binge drinking and smoking for individuals with varying intergenerational educational trajectories averaged across the relevant populations in Belarus, Hungary and Russia. The depicted results suggest that not only are there no systemic and significant risk differences in binge drinking in Belarus and Russia among individuals with different intergenerational mobility trajectories but also there are no major differences in terms of individuals’ educational attainment and this health-related behaviour. Confidence intervals for predicted probabilities of individuals with highest educational attainment in all three countries and for both genders overlap with confidence intervals of predicted probabilities for those with the lowest level of educational attainment. Nonetheless, in Hungary we observe that men in the bottom educational tertile with parents in the same level of education have 0.18 (95% CI 0.15, 0.21) probability, whereas non-mobile men in the top educational group have 0.11 (95% CI 0.08, 0.13) probability of binge drinking. Neither in the top nor in the bottom of educational tertiles are individuals’ intergenerational educational trajectories associated with their likelihood of binge drinking.

On the other hand, the estimated predicted probabilities in Figs. 3b and 4b suggest that there is a large educational gradient in smoking in all countries and for both genders and education does have significant association with this health-related behaviour. For instance, in Russia in the top educational tertile non-mobile men (PP 0.50 95% CI 0.47, 0.53) have about 10 percentage points lower risk of smoking than non-mobile individuals in the bottom educational tertile, whereas the least educated and non-mobile women in Hungary (PP 0.23 95% CI 0.21, 0.25) are 8 percentage points more likely to smoke than non-mobile women in the top educational tertile.

The effect of intergenerational educational mobility on smoking, in most instances is not statistically significant as individuals’ likelihood of smoking, when their educational attainment is accounted for, does not vary according to their trajectories of mobility. Nonetheless, for both genders in Belarus we observe a significant association between intergenerational educational mobility and smoking. First, Belarusian men with the highest educational attainment are predicted to have 0.39 (95% CI 0.34, 0.43) risk of being smokers if they did not experience intergenerational mobility, whereas this risk is only 0.29 (95% CI 0.23, 0.35) among those who moved from the lowest to the highest educational tertile. The last effect that we describe is not very large but it is still statistically significant. In terms of association between intergenerational educational mobility and smoking among women, in Belarus downwardly mobile women in the middle educational tertile have 0.05 (95% CI 0.04, 0.07) predicted probability of smoking, while upwardly mobile females are estimated to have a risk of 0.03 (95% CI 0.02, 0.04) to be current smokers. Both of these associations support the hypothesis that there are benefits of upward social mobility for health-related behaviours.

### A Summary of the Results

Before discussing the findings, we summarise the main results of our analyses. Our findings, in line with the existing literature, show that, adjusting for age, individuals’ marital status, wealth, labour market characteristics, and religious denomination are all important explanations of the considered health-related behaviours—binge drinking and smoking. In terms of the main research question of this study on the links between intergenerational social mobility and the outcome variables, we will identify those findings which were common to Belarus, Hungary and Russia and findings which were distinctive in each of these countries. In all three countries both binge drinking and smoking are more closely associated with downward than upward intergenerational mobility. Most effects for intergenerational downward mobility are manifested in relation to binge drinking, while most effects for intergenerational upward mobility are manifested in relation to smoking. One of the findings that stands out is that mobility effects of intergenerational educational attainment are more clearly manifested in Hungary (six incidence rate ratios being significant) and Belarus (four incidence rate ratio being significant) but less so in Russia (only one incidence rate ratio being significant). Furthermore, in terms of estimated predicted probabilities, the significant results are only observed in Belarus.

We have also found evidence suggesting gender differences in the links between intergenerational education mobility and health-related behaviours. First, for men four out of five statistically significant mobility effects in terms of incidence rate ratios are observed for binge drinking, while for women five out of six statistically significant mobility effects are observed for smoking. Second, for men all statistically significant effects of downward intergenerational mobility are related with the lower incidence rate ratios of binge drinking and smoking. The reverse is the case for women for whom most statistically significant consequences of downward intergenerational mobility are related to the higher incidence rate ratios of the considered health-related behaviours.

## Discussion

Political, economic and social changes in post-socialist societies during the early to mid-1990s have had profound consequences on their health-related behaviours, health outcomes and health care systems (Stuckler et al. 2009). It is likely that the collapse of social welfare regimes in these countries has intensified inequalities among various mobility groups in their access to material and other resources that are important determinants of health-related behaviours and ill-health (Mackenbach 2012). The findings presented in this study indicate that, after controlling for important socio-demographic and socio-economic confounders of binge drinking and smoking, intergenerational mobility in relative educational attainment in Belarus, Hungary and Russia does not exert systemic and consistent associations with health-related behaviours that would be expected with any single existing hypothesis. Depending on the country of analysis, the mode of mobility, the gender of respondents and the type of health-related behaviour, we find support for both the accumulation hypothesis and the Falling from Grace one, as well as those perspectives which predict positive changes in health-related behaviours with upward social mobility. One possibility is that upwardly mobile individuals, compared with non-mobile privileged groups have higher confidence and a sense of control of their own lives and follow healthy lifestyles more consistently. Upwardly mobile individuals may even express a sense of gratitude to the social environment for making the achievement of their present socio-economic advantages possible (Daenekindt et al. 2017).

As shown in the previous section, the consequences of intergenerational mobility in educational attainment on individuals’ health-related behaviours are most and least observed, respectively, in Hungary and Russia. An interesting question is what factors can explain the unique patterns in these countries. One explanation for this can be the overall differences in intergenerational social mobility between Hungary and Russia. Recent comparative research in social stratification and mobility indicates that Hungary is one of the least mobile societies in Europe, while Russia, along with other post-Soviet republics, has much higher levels of intergenerational mobility. There are reasons to believe that the effect of individual-level experience of intergenerational social mobility on health is manifested differently depending of the prevalence of social mobility in a society. Assuming that the beneficial effects of intergenerational social mobility are at least partially derived though social comparisons, then, for instance, sense of control and achievements of upwardly mobile individuals might less important if intergenerational social mobility is a widespread phenomenon. Thus experiencing social mobility Hungary might have more significant health consequences than experiencing social mobility in more fluid countries like Russia and Belarus (Bukodi et al. 2017; Gugushvili 2017a, b).

It is also important to highlight that we operationalised intergenerational educational mobility employing the “dominance approach” which used the highest level of parental education to determine intergenerational mobility. This contradicts the usual practice in public health literature that measures social mobility separately in relation to father and mother’s achieved status. Arguably, one of the main contributions of this article is that in our methodological approach we deviated from more conventional measurement of educational mobility which takes into account only absolute differences in educational qualifications between parents and their children. To compare how the latter operationalisation of intergenerational educational mobility is associated with health-related behaviours, in the unreported analysis we re-estimated associations presented in the main text, but this time with absolute measures of educational mobility.

When we switch to the absolute measure of intergenerational educational mobility we do not find any statistically significant associations in the main analysis. These findings tentatively suggest that the relative measure of intergenerational social mobility could be a more appropriate approach to study the consequences of intergenerational mobility on health-related behaviours. In the latter framework the status syndrome, as a factor in health inequalities (Marmot 2004), should be considered from an intergenerational perspective. If we assume that health-related behaviours are related to the positions that individuals possess in the social hierarchy, then intergenerational mobility must be measured in relation to change in relative position in educational distribution rather than by simple acquisition of formal qualifications by individuals in comparison to their parents. It is likely that individuals do not simply compare their status to that of their parents in absolute or relative terms, but apparently rather to their relative position among their peers than their parents’ relative position in their respective generations. It is advisable that future studies on the links between intergenerational social mobility, health-related behaviours and health consider intergenerational relative mobility instead of, or at least along with, absolute mobility measures.

One of the main findings of this study is that both individuals’ educational attainment and their intergenerational trajectories exert stronger associations with smoking than with binge drinking, especially when estimated with predicted probabilities. These findings are in line with previous research showing that intergenerational mobility has a stronger effect on daily smoking than for instance with weekly use of alcohol or being heavily intoxicated more than once a month (Glendinning et al. 1994). This is arguably not surprising, given other evidence that smoking in the home influences the likelihood of initiation by adolescents. Besides, there is a widespread, albeit controversial, view that low to moderate levels of alcohol intake might have a protective effect on health (Stockwell et al. 2016), while the healthiest level of tobacco consumption is known to be zero (Marmot 1997).

Furthermore, the observed differences in the links between intergenerational education mobility and health-related behaviours among men and women might suggest that there are gender specific manifestation of mechanisms which link mobility and specific health-related behaviours. More specifically the mechanisms predicted by *Falling from Grace* hypothesis such as elevated levels of distress seem to be are more important for women, while the accumulation perspective of the effects of mobility, which implies that individuals’ previous advantageous social conditions can protect them from various adverse behaviours, are more important for men. One of the reasons of these gender differences could be that the considered societies are still quite patriarchal and downwardly mobile men might maintain stronger links with their parents than do downwardly mobile daughters (Verdery 1994). This in turn can be manifested in more severe consequences of downward intergenerational educational mobility for women than for men.

Our study has a number of limitations. First, we detect more significant associations when the results are presented as incidence rate ratios than as predicted probabilities. The differences between relative (incidence rate ratios) and absolute (predicted probabilities) measures in social science research are a well-known phenomenon. If a relative measure is invariant to equiproportionate changes, an absolute measure is invariant to uniform changes in an outcome variable (Allanson and Petrie 2013). Therefore, deriving contradictory results is not entirely unexpected and use of only incidence rate ratios or predicted probabilities can be misleading in terms of testing hypotheses on accumulation, Falling from Grace, and positive consequence of upward intergenerational mobility (Campos-Matos and Kawachi 2015; King et al. 2012). Our analytical approach helps us not to over-interpret significant coefficients that do not show systematic patterns across different estimation measures.

The PrivMort data set, although unique in many ways, does not provide nationally representative samples and hence our findings cannot be generalised to each country’s entire populations. Data on parental education is retrospective and might be marred by respondents’ recall bias. Although earlier research does not find convincing evidence that individuals’ health-related behaviours can significantly affect their prospects of social mobility, the association between mobility and health-related behaviours may be bidirectional (see Blane et al. 1993; West 1991). Furthermore, our operationalisation of intergenerational mobility is based only on respondents’ and their parents’ educational attainment. The PrivMort data set does not include information about the measures of income, while occupational variable for respondents’ parents is only available if they were gainfully employed in the 1980s or thereafter. This does not allow us to identify if social mobility in terms of occupational social class or income has any effect on individuals’ propensity of binge drinking and smoking. Existing research suggests that various indicators of socioeconomic status, both in terms of individuals’ origins and their destinations, can have different meanings and preferably must be accounted for in future research (Bukodi and Goldthorpe 2013). Lastly, our results leave it open as to whether the exact mechanism linking intergenerational mobility in relative educational attainment, binge drinking and smoking is based on psychosocial, peer group effects or some other processes.

## Conclusion

We found that intergenerational educational mobility, measured by means of the prevalence of specified qualifications in parental and offspring generations, has varying association with binge drinking and smoking and the strength and direction of these effects depend on the country of analysis, the mode of mobility, the gender of respondents and the type of health-related behaviour. Our findings might be conditioned by the unique character of the three post-communist societies—Belarus, Hungary and Russia—which exhibit both distinct patterns of social mobility and histories of widespread binge drinking and tobacco consumption. Our tentative finding that the relative measure of intergenerational educational mobility has a stronger association with binge drinking and smoking than the absolute measure of mobility also suggests that the expansion of educational systems might have a limited effect on health-related behaviours. On the other hand, if we assume that the effect of intergenerational educational mobility on health-related behaviours is partially channelled through stress associated with individuals’ labour market circumstances, then the improvement of employment possibilities and individuals’ work environment might be a more robust way to address problems related to binge drinking and smoking.
